# Solubility Enhancement of Dihydroquercetin via “Green” Phase Modification

**DOI:** 10.3390/ijms232415965

**Published:** 2022-12-15

**Authors:** Roman P. Terekhov, Igor R. Ilyasov, Vladimir L. Beloborodov, Anastasiya K. Zhevlakova, Denis I. Pankov, Alexander V. Dzuban, Anatoliy G. Bogdanov, Georgiy N. Davidovich, Gennadii V. Shilov, Andrey N. Utenyshev, Evgenya A. Saverina, Irina A. Selivanova

**Affiliations:** 1 Nelubin Institute of Pharmacy, Sechenov First Moscow State Medical University, Trubetskaya Str. 8/2, 119991 Moscow, Russia; 2 Department of Chemistry, Lomonosov Moscow State University, Leninskiye Gory 1-3, 119991 Moscow, Russia; 3 Faculty of Biology, Lomonosov Moscow State University, Leninskiye Gory 1-32, 119991 Moscow, Russia; 4 Laboratory of Structural Chemistry, Federal Research Center of Problems of Chemical Physics and Medicinal Chemistry RAS, Acad. Semenov Av. 1, 143432 Chernogolovka, Russia; 5 Laboratory of Biologically Active Compounds and Biocomposites, Tula State University, Lenin Pr. 92, 300012 Tula, Russia

**Keywords:** dihydroquercetin, flavonoid, antioxidant, lyophilization, phase modification, solubility

## Abstract

Dihydroquercetin (DHQ) is a promising antioxidant for medical applications. The poor water solubility of this flavanonol at ambient conditions inhibits its implementation in clinical practice as an injectable dosage form. Thus, increasing water solubility is a critical step toward solving this problem. Herein we attempted to deal with this problem via DHQ phase modification while at the same time adhering to the principles of green chemistry as much as possible. Lyophilization is an appropriate method to achieve phase modification in an environment-friendly way. This method was employed to generate new phase modifications of DHQ that were then characterized. Mixtures of water with ethanol or acetonitrile were used as solvents for the preparation of the lyophilizates, DHQ_E_, and DHQ_A_, respectively. The results of dissolution testing of the obtained DHQ_E_ and DHQ_A_ demonstrated that the lyophilization increased water solubility at least 30-fold times. These new DHQ modifications were studied by scanning electron microscopy, mass-spectrometry, nuclear magnetic resonance spectroscopy, infrared spectroscopy, X-ray powder diffraction, and thermal analysis. Their solid-state phases were confirmed to differ from the initial DHQ substance without any changes in the molecular structure. Both DHQ_E_ and DHQ_A_ showed as high antioxidant activity as the initial DHQ. These data demonstrate the potential of DHQ_E_ and DHQ_A_ as active pharmaceutical ingredients for injectable dosage forms.

## 1. Introduction

Green chemistry is a relatively new area of applied science that focuses on achieving traditional chemistry goals via eco-friendly methods [1]. Its paradigm may be especially relevant for drug production, considering the ethical considerations and community demand to decrease the manufacturing process’s impact on the environment [2]. Not only remedies themselves but also their production must be safe and effective. So, “green” chemistry is the best way to stay in trend for the pharmaceutical industry.

**Figure 1:**
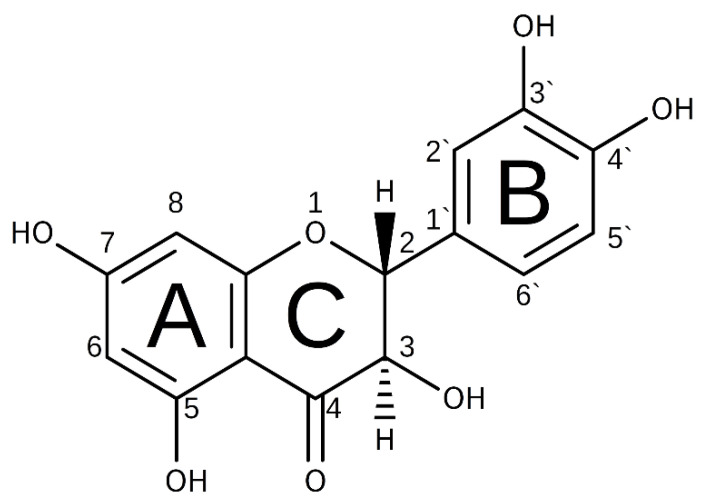
Chemical structure of 2*R*,3*R*-DHQ.

Dihydroquercetin (DHQ), also known as taxifolin, has a high potential as a drug due to multiple promising health-promoting effects. It is a natural flavanonol with the IUPAC name 2-(3,4-dihydroxyphenyl)-3,5,7-trihydroxy-2,3-dihydro-4*H*-1-benzopyran-4-one (Figure 1). This compound is widely occurring in plants [3,4,5]. The common way of the industrial production of DHQ is the extraction from the forest product residues, such as the butt log of *Larix dahurica* TURCZ [6]. This flavanonol demonstrates capillary-protective and neuroprotective effects [7,8], antitumor [9,10,11], and antiviral activity [12,13]. In Russia, it is registered as an active pharmaceutical ingredient; also, it was placed as a food supplement on the EU market.

Developing an injectable dosage form of this compound is an important step from the academic research area to clinical practice. However, this substance is poorly soluble in water at room temperature. To solve this problem, several chemical and technological approaches were applied [14,15,16]. One of the most promising ways of property optimization for DHQ is phase modification. Using the methods of crystal engineering, co-crystals of DHQ with benzaldehyde, vanillin, cinnamaldehyde, urea, and nicotinic acid were synthesized [17]. Moreover, the DHQ tubes and DHQ spheres were obtained [18,19]. All these methods result in an increase in the water solubility of this compound. However, these methods do not fully meet the principles of “green” chemistry formulated by P. Anastas and co-authors [20]. For example, benzaldehyde is toxic and explosive during storage, and spraying is a harmful production process due to the formation of fine dust.

In contrast, lyophilization, also known as freeze-drying, has several advantages that make it a favorable alternative method of phase modification. This method has a long history of use in the pharmaceutical industry [21], and, as a rule, the lyophilizates are characterized by better solubility compared with initial substances [22,23]. Moreover, freeze-drying has a solvent-saving potential [24,25]. For our data, lyophilization has not been used for phase modification of DHQ without co-formers.

So, the aim of the study was to design the “green” synthesis of new DHQ modifications with enhanced water solubility and characterize them.

## 2. Results

### 2.1. Design of “Green” Synthesis

**Figure 2:**
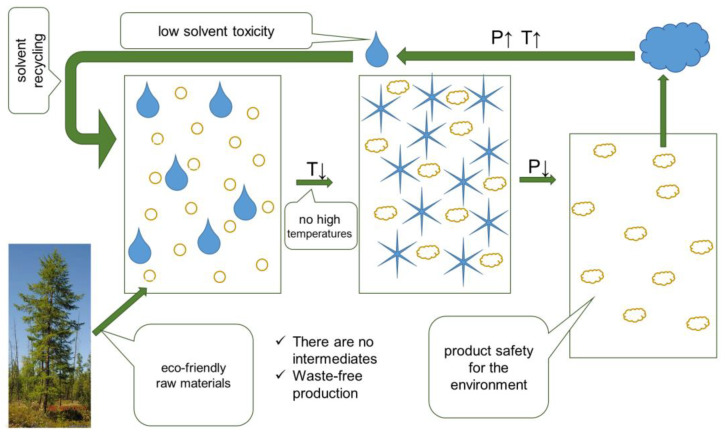
“Green” features of taxifolin lyophilization.

The principle scheme of the designed synthesis of DHQ lyophilizates is presented in Figure 2.

From a chemical point of view the following phase transition happens to initial raw DHQ (DHQ_R_) due to the formation of lyophilizates from ethanol (DHQ_E_) and acetonitrile (DHQ_A_) solutions:

(1) $$DHQ_{R}\;\overset{EtOH+H_{2}O}{\to }\;DHQ_{solution}\;\overset{T↓}{\to }\;{\left(DHQ\;+\;EtOH+\;H_{2}O\right)}_{s}\;\overset{P↓}{\to }\;DHQ_{E}+EtOH_{g}+\;H_{2}O_{g}$$

(2) $$DHQ_{R}\;\overset{MeCN+\;H_{2}O}{\to }\;DHQ_{solution}\;\overset{T↓}{\to }\;{\left(DHQ\;+\;MeCN+\;H_{2}O\right)}_{s}\;\overset{P↓}{\to }\;DHQ_{A}+MeCN_{g}+\;H_{2}O_{g}$$

where *s* is solid and *g* is gas.

The solvent may be reused in several synthesis cycles, so its mass may not be taken into account due to the calculation of process mass intensity (*PMI*) and percent atom economy (*%AE*).

Before the DHQ phase modifications, the adjustment of DHQ concentration and water: organic solvent ratio was performed. The obtained results are presented in Table 1. After optimization of the procedure, the final batches were produced. From 1.06 g and 1.08 g of DHQ_R_, 0.99 g of both DHQ_E_ and DHQ_A_ were obtained with 93.4% and 91.7% yield, respectively. There is a tendency for *PMI* to increase due to a decrease in the organic solvent concentration in the stock solution.

### 2.2. Characterization of DHQ Lyophilizates

The comparative analysis of lyophilizates was performed with the samples obtained from ethanol and acetonitrile solutions with 5% concertation of organic solvent.

#### 2.2.1. Morphology Analysis

**Figure 3:**
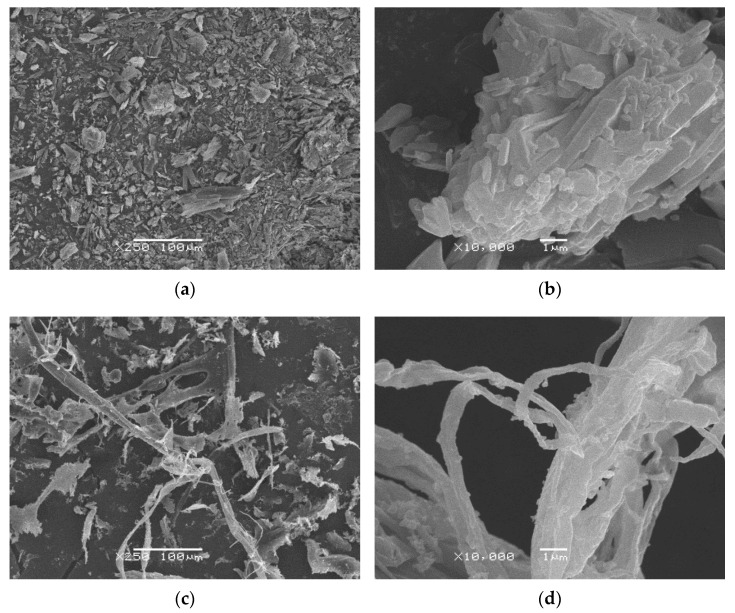

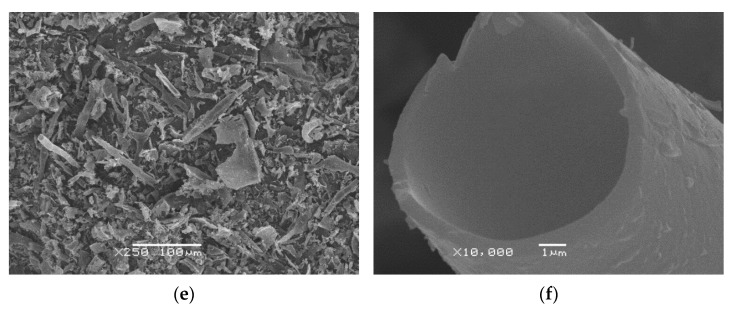
Photomicrography of different DHQ forms: (**a**) DHQ_R_ at 250× magnification; (**b**) DHQ_R_ at 10,000× magnification; (**c**) DHQ_E_ at 250× magnification; (**d**) DHQ_E_ at 10,000× magnification; (**e**) DHQ_A_ at 250× magnification; (**f**) DHQ_A_ at 10,000× magnification. The lyophilized samples were obtained from ethanol and acetonitrile solutions with 5% concertation of organic solvent.

To describe the morphology of new DHQ materials scanning electron microscopy (SEM) has been performed. Representative microphotographs show the general view of researched objects (Figure 3).

Figure 3a,b presents the morphology of DHQ_R_ under 250× and 10,000× magnifications, respectively. It can be characterized as a fine powder in which units are agglomerates. It consists of many particles that are irregular in shape. The mean diameter of agglomerates was 11.234 ± 3.215 µm.

In contrast to DHQ_R_, DHQ_E_ (Figure 3c,d) have a fiber-like morphology with a flattened oval cross-section shape. The mean size of the short dimension was 0.129 ± 0.065 µm, and the bigger one was 0.561 ± 0.065 µm. The surfaces of fibers are wrinkled and characterized by the presence of some spheroid excrescence. Its mean diameter is about 0.144 ± 0.023 µm.

The morphology of DHQ_A_ is illustrated in Figure 3e,f at different magnifications. Surprisingly, it was vessel-like with two or more enters. The mean diameter of its internal pore was about 6.959 ± 2.652 μm. At the same time, the mean thickness of its walls was 0.433 ± 0.244 µm. These external surfaces of vessels are characterized by presence of many spheroid excrescences (the mean diameter was about 0.183 ± 0.091 µm). The internal surface was smooth.

When the changes of material morphology were observed, it was necessary to confirm the saving of DHQ structure because antioxidant activity, pharmacological effects, and high safety profile of this bioflavonoid are associated with components and configurations of its molecules. To reach this goal, a complex of spectral approaches was used.

#### 2.2.2. Spectral Analysis

To characterize the molecular structure of obtained DHQ samples in comparison to the initial substance mass-spectrometry, nuclear magnetic resonance (NMR) spectroscopy and infra-red (IR) spectroscopy were applied.

Mass-spectra of analyzed DHQ samples are characterized by single peaks with *m/z* 303 (ESI, negative ionization mode).

**Figure 4:**
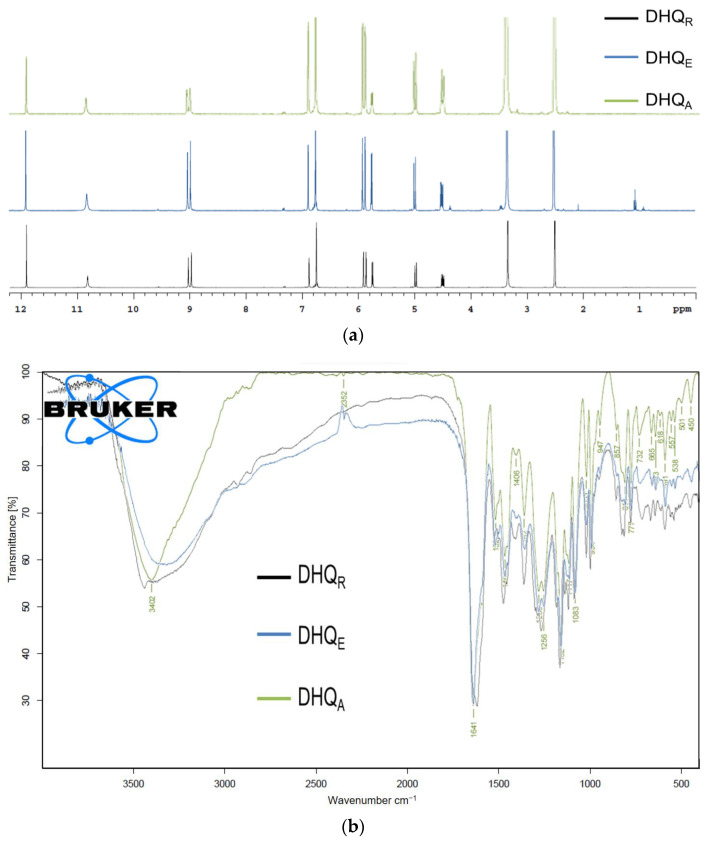
Spectral analysis of DHQ forms: (**a**) NMR ^1^H spectroscopy; (**b**) IR spectroscopy. The lyophilized samples were obtained from ethanol and acetonitrile solutions with 5% concertation of organic solvent.

NMR ^1^H spectra of different DHQ materials (Figure 4a) look similar. All three DHQ samples are characterized by singlets at 2.54, 3.33, 6.84, 8.96, 9.01, 10.80, and 11.87 ppm, doublets at 4.94, 5.73, 5.83, and 5.87 ppm, triplet at 4.47 ppm, and multiplet at 6.71 ppm. Additionally, triplet and quadruplet at 1.23 and 3.69 ppm, respectively, are observed at the NMR ^1^H spectrum of DHQ_E_.

Data from IR analysis for DHQ_R_, DHQ_E_, and DHQ_A_ showed the presence of an intensive and wide absorption band at 3400 cm^−1^ at the area of single bond stretches (Figure 4b). Moreover, at the area of double bonds, the pronounced absorption band at 1640 cm^−1^ is observed. The fingerprint regions demonstrate a similar spectrum profile. However, it is important to notice that these IR spectra are not identical: The maximum and wildness of absorption bands differ.

As it was shown that the synthesis of nanomaterial was not associated with changing of molecular formula of DHQ, it was essential to research the reasons for observed morphological differences. The answer may contain in phase transition.

#### 2.2.3. Phase Analysis

**Figure 5:**
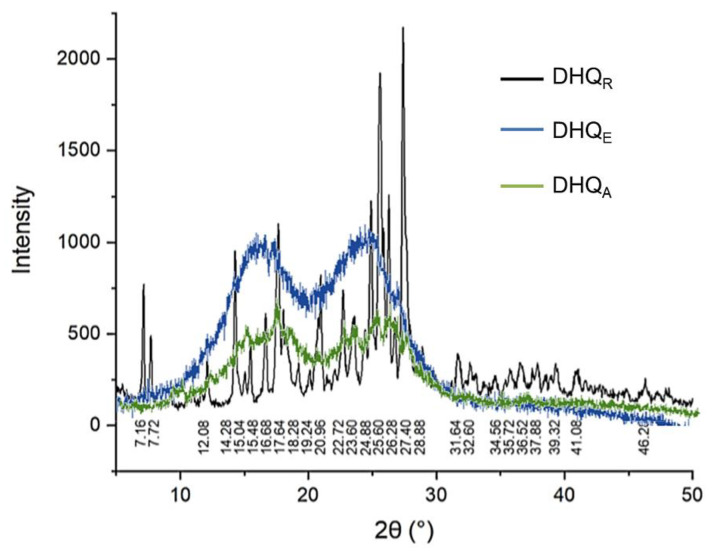
XRPD pattern of different DHQ forms at room temperature. The lyophilized samples were obtained from ethanol and acetonitrile solutions with 5% concertation of organic solvent.

The “golden standard” of phase analysis is X-ray powder diffraction (XRPD), which allows for the identification of the solids phase based on its spectrum patterns. XRPD spectrum of DHQ_R_ characterized by peaks at 2*θ* 7.16°, 7.72°, 14.28°, 15.04°, 15.48°, 17.64°, 20.96°, 24.88°, 25.60°, 26.28°, 27.40°, 31.68°, 34.56°, 37.88°, 39.32°, and 46.28° (Figure 5). In contrast, the XRPD patterns of lyophilizates are characterized by amorphous halos. The maxima of DHQ_E_ XRPD diffractogram placed at 2*θ* 15.92° and 24.50°. Moreover, DHQ_A_ is characterized by higher crystallinity than ethanol lyophilizate based on the data XRPD and its maxima moved at 2*θ* 17.20° and 25.00°.

**Figure 6:**
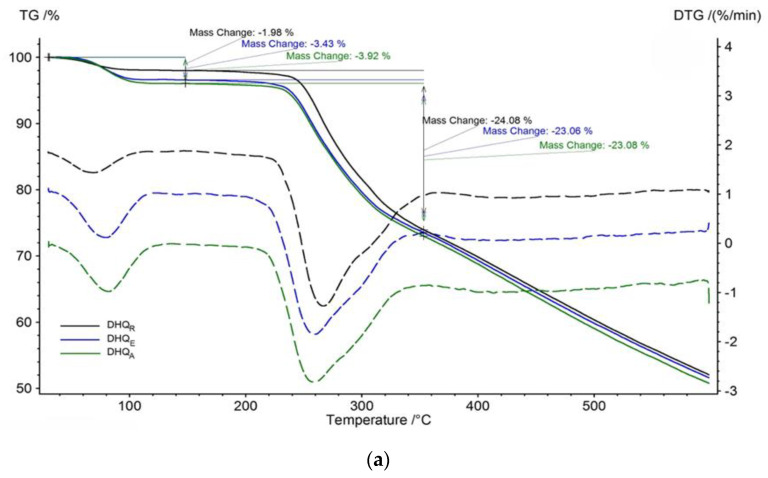

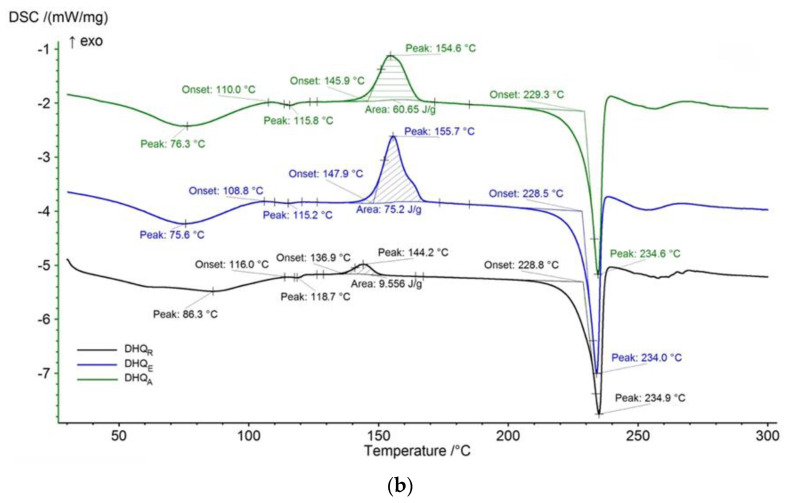
Thermoanalytical curves of different DHQ forms: (**a**) TG; (**b**) DSC. The lyophilized samples were obtained from ethanol and acetonitrile solutions with 5% concertation of organic solvent.

The thermal analysis clearly shows that all samples contain moisture in various amounts. The first step on thermogravimetric (TG) curves up to ca. 120 °C (Figure 6a), and broad endothermic effects on differential scanning calorimetry (DSC) curves up to 110–116 °C (Figure 6b) both indicate the removal of absorbed solvent (most probably pure water). All samples remain stable until ca. 230 °C, where further mass loss starts. According to DSC, they melt at 228.9 ± 0.4 °C followed by decomposition. The small endothermic effect at 109–116 °C we associate with eliminating crystal solvent from small impurities of DHQ hydrates [26]. At 137–148 °C, samples undergo some exothermic transformation, which is highly likely as cold crystallization of the present amorphous structure to a crystalline one. It agrees well with XRD results: initial crystallinity grows in the direction DHQ_E_—DHQ_A_—DHQ_R_. The larger the amorphous fraction, the greater the DSC effect.

Taken together, these data give enough information to describe the structure of new DHQ materials. Further research focused on the functional properties of lyophilizates.

#### 2.2.4. Functional Properties Analysis

Solubility is one of the key parameters to describe the biopharmaceutical characteristics of active pharmaceutical ingredients. There are different ways of solubility determination. One of them is an express method widely used in pharmaceutical science, described in European Pharmacopeia (EP). More accurate data may be obtained by the sedimentation method. Both methods were applied for DHQ samples (Table 2), and what stands out from these data is that lyophilization increased the water solubility of DHQ_E_ and DHQ_A_ compared with DHQ_R_.

The radical-cation ABTS^·+^ self-bleaching from the initial absorbance 1.1 ± 0.1 by the 6th min appeared to be 0.060 ± 0.002 and then was taken into account according to the previously reported considerations [27]. The paired Student’s *t*-test showed statistically non-significant changes of both Δ*A* and *n*-values from DHQ_R_ to DHQ_A_ and DHQ_E_ samples (Table 3).

## 3. Discussion

Despite the great potential of DHQ in medical applications, the poor water solubility of this flavanonol inhibits its implementation in clinical practice as an injectable dosage form. An increase in water solubility is critical to solving this problem. This study was conducted to optimize DHQ_R_ physicochemical properties by phase modification.

Several articles reported about phase modification of DHQ dealing with the enhancement of water solubility. However, they were associated with the application of high temperatures [19], the use of toxic solvents [28], or water gluttony without following reuse [26]. Therefore, these methods cannot be considered eco-friendly, so they have certain limitations for industrial scaling. We supposed that lyophilization could be a convenient and sustainable method for phase modification avoiding the excessive negative effects on the environment. Thus, we focused on the design of a “green” phase modification of DHQ_R_ using this method.

Directed by principles of “green” chemistry, formulated by P. Anastas and J. Warner in 1998 [29], the new DHQ materials were developed.

Prevention of pollution is a primary principle of “green” chemistry. It postulates that cleaning of products is not always an effective strategy for manufacturing. So, the number of reagents that transform not in products should be minimized. *PMI* is a recognized measurement suggested by the American Chemical Society Green Chemistry Institute Pharmaceutical Roundtable to control adherence to this principle [30]. It was shown that the key factor of *PMI* increase is solvent gluttony. The previous methods of DHQ phase modifications were characterized by the same limitation [18,26]. In contrast, lyophilization has a solvent-saving potential that decreases *PMI*, making it near to 1. The increase in this parameter may be associated with process loss.

In contrast to percent yield, which is used in traditional chemistry and demonstrates the efficacy of reaction, *%AE* is focused on the number of atoms incorporated into the final product [31]. During phase modification of DHQ via lyophilization, the only compound serves as a reactant, and the whole batch of DHQ is modified to the product. That is why *%AE* for our method is 100%. It was confirmed by a complex spectral analysis. Conversely, chemical modifications previously used to DHQ show a smaller value of this parameter [10,14,32].

Moreover, principles of “green” chemistry prescribe avoiding toxic compounds because even small amounts of toxicants may impact the environment and human health [33]. DHQ_R_, which was used as an initial compound, is waste-free and does not harm the environment [34,35]. The eco-friendliness of flavonoid materials was discussed previously [36]. In several preclinical studies, this flavonoid showed high safety profile [37,38,39]. The use of solvent is essential for lyophilization. So we focused on minimization of its hazard. Water was chosen as the main liquid component of the stock solution. To increase the solubility of DHQ and the efficacy of phase modification, the addition of organic solvents was required. For this purpose, ethanol and acetonitrile were classified as class 3 and class 2 according to FDA Guidance for Industry, respectively [40]. Five percent concentration of organic solvent in stock solution was considered a trade-off alternative between the minimization of environmental hazards and maximization of the yield of one synthesis cycle.

In general, our approach is characterized by fire safety and the recycling of solvents. Taking into account the eco-friendliness of materials on a flavonoid basis, the manufacturing of lyophilized DHQ remedies will be associated with minimal environmental hazards. Furthermore, lyophilization is widely used in the pharmaceutical industry, and the scaling of our technology is expected to be without critical difficulties.

The most important finding of the current research was the significant increase in water solubility of DHQ lyophilizates. At least a 30-fold enhancement of water solubility was observed for DHQ_E_, compared with DHQ_R_ according to EP method. The properties of DHQ_A_ were also improved by one order. At the same time, the results of sedimentation methods were considerably lower compared with the EP method. According to the sedimentation method, the solubility of DHQ_E_ and DHQ_A_ increased by 4.4 and 3.1 times, respectively.

The disagreement in solubility values obtained by different methods may be explained via the spring and parachute effect [41]. At the beginning of dissolution, the molecules of DHQ form supramolecular complexes with water. Different phase modifications generate, unlike complexes that demonstrate various solubilities that can be observed by the EP method. However, these intermolecular associates are unstable and transform into less soluble complexes later. So, the results of the sedimentation method are closer to the thermodynamically-equilibrium solubility of DHQ due to a longer time of sample processing. However, for active pharmaceutical ingredients, the concentration within the first minutes can be more relevant than after some hours, especially for the solid dosage forms and solutions prepared extempore. Further studies with more focus on the determination of the instability constant for the supramolecular complex with solvent are therefore recommended.

In fact, the EP method shows the short-term solubility of the substance, which is more pharmaceutically relevant, while in the sedimentation method, the precipitate formation is accelerated to achieve equilibrium which at ambient conditions can be substantially delayed. These findings have a certain practical sense. For DHQ_E_ and DHQ_A_, the more convenient dosage form is lyophilizate for solution preparation right before an injection compared with the industrially pre-dissolved product.

Another interesting finding was the formation of different morphologies of DHQ solid phases dependent on the solvent we used. Even though it could be guessed, no data were found on such correlations for pure flavonoid materials in the literature. At the same time, Huang et al. reported a similar behavior of diphenylalanine materials [42,43]. They suggested that the observed effect is the result of the acceptor/donor ability of the solvent, which allows it to form stable intermolecular hydrogen bonds with the diphenylalanine leading to the solvation of dipeptide molecules. It is deemed necessary to conduct more detailed experimental and theoretical studies for a better understanding of the solvent influence on the nature of the DHQ assembly and directing the self-organization of its solid phase.

To describe the morphology of DHQ substances, SEM was performed. In general, the structure of DHQ_R_ correlated with the results of the previous research [18,44]. As this material did not demonstrate a particle size of less than 0.100 µm in any dimension, it could not be considered a nanomaterial according to IUPAC nomenclature [45]. At the same time, DHQ_E_ microstructure may be identified as “nanoplate,” as one of the fibers dimensions did not differ substantially from 0.100 µm. Additionally, DHQ_A_ may be classified as a “nanostructured material”, because its surface is coated with nanostructured inclusions. Up to 98.1% of this material’s volume is a cavity. So, DHQ_A_ may find application in pharmaceutical science not only as an active pharmaceutical ingredient but also as a carrier for other compounds [46,47].

The goal of complex spectral analysis for DHQ lyophilizates was to confirm the absence of molecular structure changes in flavanonol molecules during the phase modification processing. The same 303 *m/z* signals were observed in the negative ion mode ESI-MS, corresponding to a quasi-molecular ion of DHQ, whose molecular mass is 304 g/mol [19,48]. The NMR ^1^H spectra signals correlate with the spectral characteristics of DHQ described before [49]. There are singlets at 2.54 ppm and 3.33 ppm, which associate with the DMSO and water, respectively. The major compound of all analyzed samples was identified as a 2*R*,3*R*-stereoisomer of DHQ [50]. Triplet at 1.23 ppm and quadruplet at 3.69 ppm in the spectrum of DHQ_E_ show the presence of residual ethanol, which was used in the synthesis of this sample. Based on its intensity, the quantity of this solvent was less than 2.0%. The signal of acetonitrile protons at 2.07 ppm was absent, so this solvent was completely removed from the sample. The patterns of IR spectra of all analyzed DHQ forms confirmed the presence of all characteristic structural fragments: hydroxyls, carbonyl group, and aromatic rings. To sum up, these indicate no new covalent bond formation and confirm that the molecular structure of DHQ remained unchanged.

Despite the absence of molecular structure changes, the observed modification of morphology and physicochemical properties may be explained by forming a new solid phase. To confirm this, the XRPD and thermal analysis were applied. XRPD pattern of DHQ_R_ shows it is a crystal material and demonstrates a good correlation with previous studies of DHQ_R_ [18,51]. Both DHQ_E_ and DHQ_A_ are amorphous materials. Previously we described another DHQ amorphous material characterized by spherical morphology and amorphous halo in the XRPD pattern with only one maximum at 2*θ* 24.92° [19]. This result differs from the findings presented here, as diffractograms of DHQ_E_ and DHQ_A_ showed two maxima each and were not identical. Furthermore, DHQ_A_ shows more crystallinity in contrast to the smoother pattern of DHQ_E_. This suggestion is supported by the results of thermal analysis. The exothermic effect at 155.1° can be associated with the cold crystallization of an amorphous structure to a crystalline one. According to DSC data, in DHQ_A_, this process is less intensive than the DHQ_E_, which may explain the higher crystallinity of this material. Moreover, it can be noted that all three analyzed DHQ samples contain the same moisture that is completely released at 110 °C, and at 234.5 °C, the melting proceeds with the decomposition of flavonoid. Tacking together these data suggest that DHQ_E_ and DHQ_A_ are new amorphous forms of this flavonoid, and according to pharmaceutical regulatory documents, they can be identified as polymorphic modifications of DHQ_R_ [52].

The theoretical considerations with respect to the relationship between the modification of solid forms of substance and its antioxidant capacity seem obvious and difficult to argue: No matter which solid form was had, after dissolution, only the concentration matters (of course, in case the obtained solution is a true solution). This makes the comparison of the antioxidant capacity of different forms redundant. However, in some works, the antioxidant capacity was not only measured but also demonstrated a huge difference [53]. Our attempts to find such a difference failed: As we expected, all the DHQ samples demonstrated a rather high antioxidant capacity with no significant difference between the analyzed samples. Apparently, those who discover this enhancement in the antioxidant capacity of modified solid forms (with no chemical changes) should ascribe them to the different amounts of the dissolved fraction and, therefore, different concentrations of the obtained supernatant solutions. Different concentrations demonstrate different antioxidant capacities, which cannot be attributed to the specific properties of the modified forms without robust evidence. The latter point also makes the dissolved fraction concentration measurements much more reasonable than their antioxidant capacity.

These data primarily suggest that during lyophilization, DHQ solubility rises its solubility without losing its antioxidant activity. As a result, thanks to the phase modification the bioavailability may be increased, so the concentration of the active pharmaceutical ingredient in blood will also grow. In our previous work, we showed that higher bioavailability of DHQ is associated with a more pronounced pharmacological effect [54]. In the case of DHQ lyophilizates, the same behavior is expected. Regarding the fast decrease of DHQ_E_ solubility after dissolution, the lyophilized injection would be the most optimal formulation for future remedies on a DHQ basis.

## 4. Materials and Methods

### 4.1. Materials

DHQ_R_, used as the initial compound for phase modification and as a comparison object, was purchased from Ametis JSC (99.1%, Blagoveshchensk, Russia).

ABTS, 2,2′-azinobis(3-ethylbenzothiazoline-6-sulfonic acid) diammonium salt, potassium persulfate (di-potassium peroxydisulfate, PP), and the components for the preparation of the phosphate buffer saline pH 7.0 (PBS)—i.e., disodium hydrogen phosphate, sodium dihydrogen phosphate, and sodium chloride—were obtained from Sigma-Aldrich (Burlington, MA, USA).

Acetonitrile (HPLC-grade, Merck Ltd., Darmstadt, Germany), denatured ethanol (99.8%, Carl Roth GmbH, Karlsruhe, Germany), and distilled water were used as solvents. Ethanol (HPLC-grade) and deionized water in the antioxidant capacity assay were from Merck Ltd. (Darmstadt, Germany) and LLC Smoly (Moscow, Russia), respectively.

### 4.2. Lyophilizates Preparation

To generate a stock solution of DHQ_E_ and DHQ_A_, 1.0 g of DHQ_R_ was dissolved in 50 mL of denatured ethanol and acetonitrile, respectively. The organic solutions were diluted with distilled water up to 1.0 L.

The stock solutions were frozen immediately at −78 °C in dry ice for 24 h. The solid mixture was connected to the laboratory freeze dryer Alpha 1–2 LD (Martin Christ Gefriertrocknungsanlagen GmbH, Osterode am Harz, Germany), which operated at 35,463.75 Pa and −55 °C for 36 h.

### 4.3. Assessment of Synthesis Eco-Friendliness

*PMI* is calculated by Equation (3):

(3) $$PMI=\frac{m_{total}}{m_{product}},$$

where *m_total_* is the mass (kg) of all materials, used in the process or step, and *m_product_* is the mass (kg) of the product.

*%AE* is obtained by Equation (4):

(4) $$\%AE=\frac{M_{product}}{\sum M_{reactant}}\times 100\%,$$

where *M_product_* is the formula weight of the product (g/mol) and *M_reactant_* is the formula weight of all reactants (g/mol) of the chemical process.

### 4.4. SEM

The analyzed samples were fixed on a sample holder using double-sided carbon tape, and at the next step it, was coated with gold in Argon atmosphere at 13.33 Pa in an IB-3 ion coater (Eiko Engineering Co., Tokyo, Japan).

Scanning electron microscope JSM-6380LA (JEOL Technics LTD, Akishima, Japan) was used for imaging under ×250 and ×10,000 magnifications. The microscope was operated at 20 kV accelerating voltage in secondary electron imaging mode.

The gold coating was approximately 20 nm thick. So, the metal layer thickness influence was negligible.

### 4.5. Mass-Spectrometry

Mass spectra were recorded with Advion LC-MS (Advion, Ithaca, NY, USA) in 0.1% formic acid methanol-aqueous solution (4:1, volume ratio). Mass-spectrometer was equipped with an electrospray ionization source and operated in negative ion mode.

### 4.6. NMR ^1^H Spectroscopy

NMR ^1^H spectra were recorded with a Varian VNMRS-400 (Agilent, Santa Clara, CA, USA) at ambient temperature in DMSO-*d_6_* solutions. Chemical shift values are given in *δ* scale relative to tetramethylsilane.

### 4.7. IR Spectroscopy

IR spectra were recorded with a Bruker ALPHA-T FT-IR spectrometer (Bruker Corporation, Bremen, Germany) in KBr pellets.

### 4.8. XRPD

XRPD patterns were recorded with an ARL X’TRA X-ray diffractometer (Thermo Electron Corporation, Waltham, MA, USA) that was equipped with a vertical *θ*–*θ* wide-angle goniometer and a Peltier solid-state detector with monochromatic Cu-Kα radiation (*λ* = 1.54 Å). It was operated at 22 °C, 25 mA, and 45 kV. The integration time was 1 s.

### 4.9. Thermal Analysis

Thermal analysis was carried out on DSC 204 F1 Phoenix^®^ differential scanning calorimeter (NETZSCH, Selb, Germany) and TG 209 F1 Libra^®^ thermobalances (NETZSCH, Selb, Germany). Samples weighing 2.00–10.00 mg were tested in aluminum (DSC) and alumina (TG) crucibles (lid with a hole) under dry nitrogen flow (20–70 mL·min^−1^) with a heating rate of 10 °C·min^−1^. Instruments were previously calibrated for temperatures and enthalpies of phase transitions of pure (99.999%) standard substances in compliance with ASTM Practices E967, E968, E1582, and E2253: cyclohexane, Hg, Ga, benzoic acid, In, Sn, Bi, Pb, Zn, CsCl—for DSC; In, Sn, Bi, Zn, Al, Ag, Au—for TG. Calcium oxalate monohydrate was used for the validation of thermobalances. Experimental data were processed in NETZSCH Proteus^®^ Software according to ASTM E794, E2550, and ISO 11357-1.

### 4.10. Solubility

#### 4.10.1. EP Method

The analysis was performed in accordance with the pharmacopoeial monograph of European Pharmacopeia 10.0 [52] at ambient conditions.

#### 4.10.2. Sedimentation Method

To determine the concentration of DHQ solutions, UV spectrometry was applied. Three hundred mg of each DHQ sample were dissolved in 10.0 mL of ethanol and centrifuged for 5 min at 10,000 rpm by centrifuge MiniSpin^®^ (Eppendorf AG, Hamburg, Germany). The analyzed solutions were prepared by dilution of the supernatant 50 μL up to 25 mL with distilled water. The UV spectra were recorded with a Cary-100 (Varian, Palo Alto, CA, USA). The accurate concentrations were calculated using a calibration curve.

### 4.11. Antioxidant Capacity

To compare the antioxidant capacity of DHQ in different forms, we employed the ABTS/PP assay, as reported by Re et al. [55], with some modifications [24,56]. In brief, an antioxidant was added to the pre-generated stock solution of radical-cation ABTS^•+^, and then, after 6 min incubation in the dark place at 32 °C, the decrease in the absorption at 730 nm was calculated. ABTS^•+^ self-bleaching after 6 min was measured in the same conditions without the addition of antioxidants. The initial ABTS^•+^ absorbance in the incubation mix before the addition of an antioxidant was adjusted to 1.1 ± 0.1. The stock solution of the radical-cation ABTS^•+^ was prepared by the incubation of the ABTS with PP at final concentrations of 7 mM and 2.5 mM, respectively, during 12–16 h in the dark. UV-Vis spectra were obtained on a Cary 100 (Varian, Palo Alto, CA, USA).

The absorbance decrease ∆*A* at 730 nm due to inhibition of radical-cation ABTS^•+^ was calculated as follows (5):

∆*A* = *A*_0_ − *A*_1_ − ∆*A_sb_*, (5)

where *A*_0_ and *A*_1_ are the ABTS^•+^ absorbances at 730 nm, pathlength 1 cm, before and 6 min after the antioxidant addition, respectively. The self-bleaching of radical-cation ABTS^•+^ ∆*A_sb_* was calculated as the difference between the ABTS^•+^ absorbance right after the addition of stock ABTS^•+^ to PBS to give absorbance 1.0 ± 0.1 and 6 min later.

The amount of ABTS^•+^ radicals scavenged by one molecule of antioxidant (*n*-value) was calculated according to the Equation (6):

(6) $$N=\frac{C_{ABTS\cdot +}}{C_{antioxidant}}=\frac{\Delta A}{\varepsilon \;\cdot \;C_{antioxidant}},$$

where *C_ABTS_*^•+^ is the concentration of the scavenged ABTS^•+^, *ε* is the extinction at 730 nm 15,000 L·mol^−1^·cm^−1^, *l* is the optical pathlength 1 cm, and *C_antioxidant_* is the concentration of the antioxidant.

### 4.12. Statistics

The results are shown as the mean (± standard deviation) of at least three determinations. The paired Student’s *t*-test was used for the analysis of experimental results.

## 5. Conclusions

This project was undertaken to design the “green” synthesis of DHQ lyophilizates and evaluate these properties. The investigation of DHQ_E_ and DHQ_A_ has shown that these substances are amorphous nanostructured materials. So, the new material may be considered as new polymorphic modifications of the initial DHQ_R_. Phase modification via lyophilization changed neither the molecular structure of flavonoids nor their antioxidant activity. However, one of the most important findings from this study is that freeze drying increased the water solubility of DHQ at ambient conditions. These data suggest that DHQ_E_ and DHQ_A_ can be used as active pharmaceutical ingredients for injectable dosage forms. A natural progression of this work is to develop the validated technology of standardized DHQ lyophilizate products that would be appropriate for the industry. Further research is needed to determine the instability constants for DHQ solvate complexes and to research the pharmacokinetics of remedies on this flavonoid basis in the injections.

## Figures and Tables

**Table 1:** Results of synthesis.

Solvent	Water: Organic Solvent Ratio	Yield, %	*PMI*	*%AE*, %
Ethanol	85:15	none *	-	-
	90:10	none *	-	-
	95:5	93.4 ± 1.1	1.07	100
Acetonitrile	85:15	97.0 ± 1.9	1.03	100
	90:10	95.6 ± 1.4	1.05	100
	95:5	91.7 ± 1.1	1.09	100

* The boiling without sublimation takes place.

**Table 2:** Solubility of DHQ substances.

Sample	Term by EP	Solubility, mg/mL	
		By EP Method	By Sedimentation Method
DHQ_R_	very slightly soluble	[0.1–1.0]	0.70 ± 0.02
DHQ_E_	soluble	[100.0–30.0]	3.09 ± 0.09
DHQ_A_	slightly soluble	[1.0–10.0]	2.14 ± 0.06

The lyophilized samples were obtained from ethanol and acetonitrile solutions with 5% concertation of organic solvent.

**Table 3:** Antioxidant capacity of the DHQ samples.

Sample	Antioxidant Capacity	
	Δ*A*	*n*-Value
DHQ_R_	0.511 ± 0.017	7.30 ± 0.24
DHQ_E_	0.504 ± 0.026	7.19 ± 0.37
DHQ_A_	0.499 ± 0.014	7.13 ± 0.19

The lyophilized samples were obtained from ethanol and acetonitrile solutions with 5% concertation of organic solvent.

## Data Availability

Not applicable.
